# Nanoscopic imaging of thick heterogeneous soft-matter structures in aqueous solution

**DOI:** 10.1038/ncomms12729

**Published:** 2016-09-06

**Authors:** Tobias F. Bartsch, Martin D. Kochanczyk, Emanuel N. Lissek, Janina R. Lange, Ernst-Ludwig Florin

**Affiliations:** 1 Center for Nonlinear Dynamics, Physics Department, The University of Texas at Austin, 2515 Speedway, Austin, Texas 78712, USA; 2 Howard Hughes Medical Institute and Laboratory of Sensory Neuroscience, The Rockefeller University, 1230 York Avenue, New York, New York 10065, USA; 3 Biophysics Group, Department of Physics, Friedrich-Alexander-University of Erlangen-Nürnberg, Henkestrasse 91, 91052 Erlangen, Germany

## Abstract

Precise nanometre-scale imaging of soft structures at room temperature poses a major challenge to any type of microscopy because fast thermal fluctuations lead to significant motion blur if the position of the structure is measured with insufficient bandwidth. Moreover, precise localization is also affected by optical heterogeneities, which lead to deformations in the imaged local geometry, the severity depending on the sample and its thickness. Here we introduce quantitative thermal noise imaging, a three-dimensional scanning probe technique, as a method for imaging soft, optically heterogeneous and porous matter with submicroscopic spatial resolution in aqueous solution. By imaging both individual microtubules and collagen fibrils in a network, we demonstrate that structures can be localized with a precision of ∼10 nm and that their local dynamics can be quantified with 50 kHz bandwidth and subnanometre amplitudes. Furthermore, we show how image distortions caused by optically dense structures can be corrected for.

## Abstract (web summary)

Integration time limits the capacity of super-resolution microscopy to study dynamics. Here, Bartsch *et al*. use the 3D scanning of a nanoparticle held in an optical trap, and its thermal noise motion within the trap, to image the local dynamics of soft-matter structures with a 50 kHz bandwidth.

## Graphical abstract

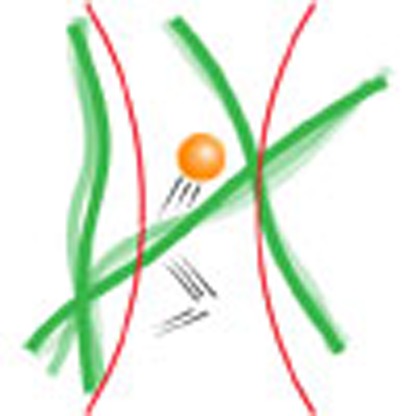

For decades, resolving the three-dimensional ultrastructure and organization of cells, or soft matter in general, has been limited to electron microscopy, which requires special preparation of samples and is typically not compatible with live-cell observation. However, recently the diffraction barrier has been overcome by super-resolution fluorescence microscopes such as stimulated emission depletion microscopy (STED)^1^, photoactivated localization microscopy (PALM)^2^ or stochastic optical reconstruction microscopy (STORM)^3^, which provide specific molecular contrast on fixed samples in aqueous solution. The focus is now shifting to the observation of dynamics in living cells, and surprising progress has been made, for instance vesicle motion in *Drosophila* larvae and the uptake of viral particles has recently been resolved by ultrafast STED nanoscopy with 5–10 ms temporal resolution^4^.

The resolution in lens-based super-resolution microscopes is limited by the number of photons detected from fluorophores and by the density of the fluorescent labels. For the observation of dynamics, however, the photon rate is the limiting factor, since the precision in localizing a fluorophore depends on the number of photons that can be collected as well as on the motion of the labelled structure, which limits the time available to collect the photons. However, the current discussion in literature concerning the requirements for molecular resolution imaging of live cells or, more generally speaking, of soft-matter structures, does not address the fundamental aspect of thermal motion in soft and porous media. High precision in label localization and consequently high resolution of soft structures can be achieved only with sufficiently short detector integration times. Even for the fastest reported camera integration times of 2 ms in STORM^5^ and 1 ms in STED^4^, a 10 nm fluorescent particle in water at room temperature will diffuse on average about 0.8 and 0.5 μm, respectively. Although one can precisely calculate the center position of the motion-blurred broad spot on the camera, the true position of the particle is not measured with high precision unless it is immobilized on a very rigid structure. This problem is often solved by fixation of the sample by chemical crosslinkers, which increases the sample's rigidity, and by subsequent labelling of the sample with fluorescent antibodies. In this case, fluorescent probes are rigidly coupled to stiff structures, enabling fluorescence-based super-resolution imaging. However, such fixation necessarily prevents the measurement of mechanical properties of the observed matter due to crosslinking and dense antibody loading, and rules out imaging of living cells. In unfixed samples, both immobilized and freely diffusing probe particles are special cases, since most molecules in a living cell are restricted in their motion either by soft fluctuating structures that surround them or by being part of such structures. The resulting complicated three-dimensional thermal motion of objects, including fluorescent labels, reduces the localization precision in super-resolution microscopy. Furthermore, image distortions due to optical inhomogeneities are mostly ignored in super-resolution microscopy or minimized by working with extremely thin samples^6^. Such optical inhomegeneities are known to seriously degrade imaging performance under all but the most ideal conditions^7^.

Here we focus on the motion blur that occurs when probes are freely diffusing and/or are bound to rapidly diffusing structures in complex three-dimensional confinements. Fluorescence-based super-resolution methods are unable to precisely localize such rapidly moving probes unless they are confined or slowed down by a viscous medium. We show that rather than limiting precision, thermal motion of such rapidly diffusing probe particles can be used to gain structural information provided that the restriction to fluorescent labels is dropped, and instead highly scattering nanospheres are used, which can be tracked in three dimensions with short integration times and hence with negligible motion blur. We also gain insights into the mechanics of the imaged structures and into their interaction with the probe particle. Equally important, our approach allows studying and correcting for distortions in the local geometries of the imaged structures due to optical inhomogeneities. Our thermal noise imaging method uses a single diffusing nanoparticle to image soft porous nanostructures. The probe's three-dimensional motion reveals the local geometry with a precision of a few nanometres. As a true three-dimensional imaging technique, our method does not directly compete with atomic force microscopy, which is able to achieve atomic resolution but can only image the topology of surfaces well oriented with respect to the probe. The same limitation applies to other novel forms of scanning probe microscopy, for example, surface imaging by cigar-shaped probes held in holographic optical tweezers^8^ or near-field scanning optical microscopy (NSOM/SNOM) by light emitting rod-shaped probes^9^.

We apply our method to microtubules and to a network of thick and optically dense collagen fibrils and demonstrate how to correct for image distortions caused by light–fibril interaction. Finally, we find that even these thick filaments, which are usually considered to be rigid, fluctuate on a length scale of 10 nm within a frequency band of up to 1 kHz, underlining the need for fast position measurements for reliable imaging of soft nanostructures.

## Results

### Principle of the experiment

**Figure 1:**
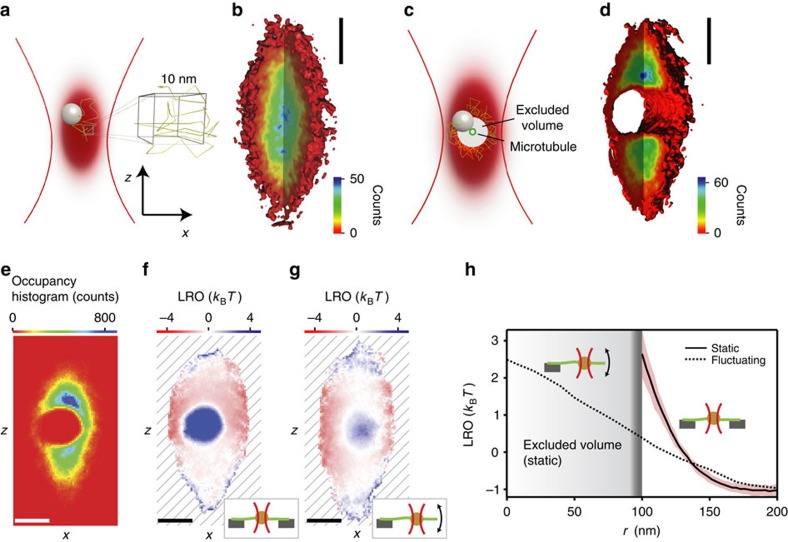
Principle of thermal noise imaging. (**a**) The trapping volume is subdivided into voxels (10 nm side length) to calculate the position histogram. (**b**) Isosurface (red) of equal voxel occupancy. The heat map of the cut-open section indicates the occupancy of each voxel. (**c**) If a filament (green circle) is introduced into the optical trap, parts of the trap become inaccessible. The excluded volume depends on the filament and particle radius. (**d**) Isosurface of equal voxel occupancy for an optical trap intersected by a microtubule confined by rigid supports on both ends (see inset in **f**). Both isosurfaces are drawn for an occupancy of 2 counts. (**e**) Two-dimensional projection of the position histogram along the filament axis. (**f**) Two-dimensional logarithmic relative occupancy (LRO, see main text) calculated from the position histogram shown in **e**. The LRO is a measure for the interaction energy between the probe and the imaged filament. Voxels that the probe can never visit (excluded volume) have an interaction energy of infinity (dark blue). The hatched area indicates the space inaccessible to the particle due to the confinement by the optical trap. The inset indicates our assay: a microtubule (green) is spanned over a carbon grid (grey) and is imaged by a probe particle (orange) confined by weak optical tweezers (red). (**g**) Two-dimensional LRO for a probe particle interacting with a microtubule immobilized on one end, while the other end is free to laterally fluctuate driven by thermal forces (see inset). The LRO shown was acquired ∼2.8 μm from the microtubule's fixed end. The filament's thermal motion allows the probe to visit voxels that were previously inaccessible (**e**,**f**), and the excluded volume disappears. (**h**) The radial profile (see Methods) of the static microtubule's LRO (solid line) reveals the extent of the excluded volume, which is made up of all voxels that were inaccessible to the diffusion of the probe. The increase in interaction energy between probe and filament for separations shorter than 150 nm is due to an interplay of electrostatic and steric forces and the thermal motion of the filament. The region of uncertainty indicates the standard deviation of 33 independent recordings of the same filament. The radial LRO for the fluctuating microtubule (dashed line) penetrates into the previously excluded volume. Its diminished slope is a result of the much broader spatial probability density of the fluctuating microtubule. Scale bars, 200 nm.

Thermal noise imaging was introduced more than a decade ago^10^, but it has until now remained challenging to turn it into a quantitative method for three-dimensional imaging of soft nanostructures. Thermal noise imaging exploits the thermal motion of a nanoparticle as a natural scanner, similar to a protein searching for a binding site. However, in contrast to a freely diffusing protein, the probe particle is confined by an optical trapping potential that forces it to revisit the same volume over and over until its local surroundings have been sufficiently sampled. The laser light that generates the optical trap serves simultaneously as an illumination source for the position detection; hence the position measurement precision is no longer limited by photon statistics. With current detectors, a single position can be measured in three dimensions with a megahertz bandwidth^11^,^12^. Within this microsecond integration time the particle can only diffuse a few nanometres (thus avoiding motion blur) and up to a million of positions per second can be measured continuously. This allows dense sampling of the accessible space in which objects are represented by the excluded volume they cause (Fig. 1).

### The precision of thermal noise imaging

To demonstrate the precision with which structures can be localized using thermal noise imaging, we imaged a 25 nm diameter microtubule supported on both ends by a carbon grid. The tube is formed by protofilaments that are held together by weak lateral interactions. They are the stiffest member of the cytoskeleton; the persistence length is typically a millimetre^13^. For our experiment, as long as they are confined on both ends and are no longer than a few micrometres, we can consider them as rigid rods with a well-defined diameter.

In a typical thermal noise imaging experiment, the probe particle is held by optical tweezers next to a structure of interest and its position is recorded with high bandwidth for multiple seconds. A three-dimensional position histogram is then calculated from the millions of position measurements and displayed as an isosurface (Fig. 1). Without any object nearby, the particle explores the entire trapping potential and the isosurface has a cigar-like shape, as expected for a three-dimensional harmonic trapping potential^14^. The slice through the histogram shows that positions at the center of the trapping potential are most populated, in agreement with Boltzmann statistics. But when the center of the trapping potential is moved to the microtubule axis, the probe particle has to diffuse around it, leaving a cylinder shaped excluded volume with a diameter that is approximately the sum of the diameter of the probe particle plus the filament.

Finding the cylinder axis from a thermal noise image is more involved because the position of the isosurface depends on the position histogram, which in turn not only depends on the geometry of the filament, but also on the shape of the trapping potential and its relative position to the filament. This leads to distortions of the geometry as shown in Fig. 1e. To correct for the influence of the trapping potential, we introduce the logarithmic relative occupancy (LRO)


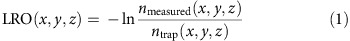


where *n*_measured_ is the measured position distribution of the tracer particle and *n*_trap_ the distribution expected for the trap without an object. The LRO is essentially a measure of how the presence of an object modifies the histogram of a trapped particle. A voxel that is unperturbed by the presence of an object will have a LRO value of zero, excluded volumes will have a value of positive infinity, and voxels with increased probability will have negative values. However, the LRO is more than that: the LRO reflects the interaction potential of the particle with the scanned structure measured in units of the thermal energy *k*_B_*T* under the condition that the entire volume was sampled long enough so the histogram approaches the equilibrium distribution. Figure 1f shows the LRO for the histogram in Fig. 1e. The presence of the microtubule causes a circular excluded volume (infinite LRO) and forces the particle to explore the voxels around the microtubule with increased probability, resulting in a negative LRO for the filament's surroundings. The projection is now radially symmetric and we find the position of the axis by fitting a Gaussian distribution to the center of the LRO. The resultant uncertainty for the center position by the fit is less than a nanometre. The very small uncertainty is a result of the large number (*n*=400,000) of local position measurements around the filament. The position of the cylinder axis was determined from 33 histograms recorded at the same position of a microtubule. After correcting for a small instrument drift, the uncertainty of the cylinder axis was 7 nm in the *x* and *y* direction and 15 nm in the *z* direction, which shows that a filament can be localized with <10 nm uncertainty in the lateral plane. The increased uncertainty along the optical axis was a result of the wider axial trapping potential and the consequently lower number of position measurements near the filament.

### Imaging immobile and fluctuating structures

Next, to model a soft, fluctuating structure, we immobilized a microtubule on one of its ends, while leaving its other end free to thermally fluctuate (Fig. 1g). As the filament fluctuates transverse to its long axis, the probe can now visit voxels that were previously inaccessible, and the excluded volume disappears. The particle now probes the filament's spatial probability density: the LRO has its maximum at the microtubule's most likely position and slowly decays with increasing radial extension (Fig. 1h, dashed line).

We analysed the radial behaviour of the immobile double-confined microtubule's LRO as a function of the distance from the filament axis. Figure 1h shows the average radial LRO (solid line) calculated around the cylinder axis position for 33 consecutive measurements of the same microtubule at the same position. The red shading represents the s.d. for each point on the curve (Methods). The radial LRO represents the interaction potential of microtubule and probe particle; this potential depends on the radius of the microtubule, its thermal fluctuations, the radius of the probe particle, and the thickness of the passivation layer around the particle (Methods). From the maximum of the LRO, we find a radius of the excluded volume of 100±15 nm (error defined in Methods). The expected radius of the excluded volume is the sum of the bead radius (95±15 nm), the radius of the microtubule (12.5 nm), and ∼5 nm thickness of the passivation layer around the particle, which consisted of a poloxamer coating. The resulting radius of 112 nm agrees within the uncertainties with the 100 nm radius from the LRO analysis. The uncertainty in the estimate of the excluded volume is dominated by the uncertainty of the probe radius, but it should be possible to reduce this uncertainty to a few nanometres using an *in situ* measurement method for the particle radius^15^.

### Imaging thick filaments in heterogeneous media

The high-bandwidth detection of the probe particle's position by forward scattered laser light relies on the probe's higher index of refraction relative to its environment and scales with the radius of the probe, to the 6th power for a Rayleigh scatterer. For optically dense filaments (Supplementary Fig. 1) with diameters on the order of the diameter of the probe particle, one expects major distortions of the detector signal because the filament scatters light in addition to the light scattered by the probe. A second problem is that thicker filaments cannot be imaged with a single-position histogram of the probe's confined motion because the corresponding excluded volume exceeds the size of the trapping volume and thermal diffusion is not sufficient to drive the probe around the filament. To test whether thermal noise imaging can be used in thick heterogeneous media, we studied collagen I networks as a model system. Collagen I networks under our conditions (Methods) are built of fibrils with mesh sizes of 2.6±0.1 μm (see ref. 16). Fibrils have an estimated persistence length of several metres^17^ and thus can be considered for our purposes to behave as rigid rods, much like the microtubules in the previous example.

**Figure 2:**
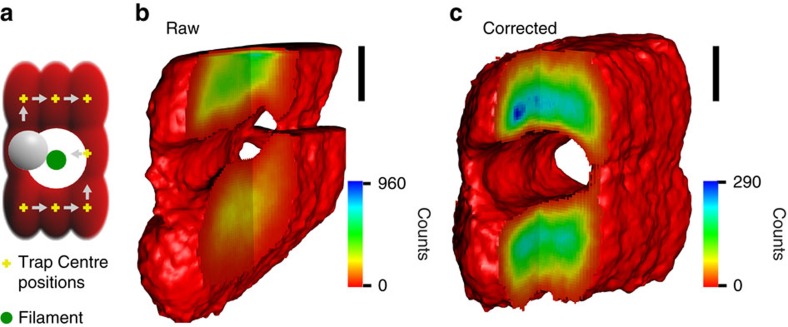
Thermal noise imaging of a thick and optically dense filament. (**a**) Since thick filaments cannot be imaged by individual trapping volumes, the center position of the optical trap is instead moved on a grid and individual thermal noise images at each grid position are acquired and subsequently combined to yield a single large image. (**b**) Combined thermal noise image of a collagen fibril that has not yet been corrected for light scattered by the optically dense filament. (**c**) Same data as **b**, but corrected for light scattered by the imaged fibril (see Methods). Isosurfaces are shown at 10 counts. Scale bars, 200 nm.

Scanning of larger volumes can be achieved by displacing the optical trap along a three-dimensional grid that fills a region of interest while recording a thermal noise image at each grid point (Supplementary Fig. 2). Adjacent points are spaced so that their thermal noise images overlap. Local thermal noise images are then combined into a single global position histogram. As expected, however, without correcting for the light scattered by the collagen fibrils, the geometry of the long-range imaged collagen filament is distorted (Fig. 2). To correct for the light scattered by the fibrils we considered the detected intensity to be the sum of the signal caused by the network alone plus the position signal of the particle in absence of the network (Methods and Supplementary Fig. 3). Then if the detector signal caused by fibrils alone is known, the true position of the probe particle can be calculated by subtracting the network signal from the total signal. The network thus gives rise to a time-independent offset signal, which has to be removed from the total signal on the detector. In the following we call this ‘offset correction'. The offset signal can be recorded by scanning the focused laser beam through the network without a trapped probe particle. Figure 2c shows a thermal noise image after correcting for the network signal. In contrast to the raw thermal noise image (Fig. 2b), the filament appears now as a uniform cylinder, indicating that the simple correction algorithm accounts for most of the image distortions.

**Figure 3:**
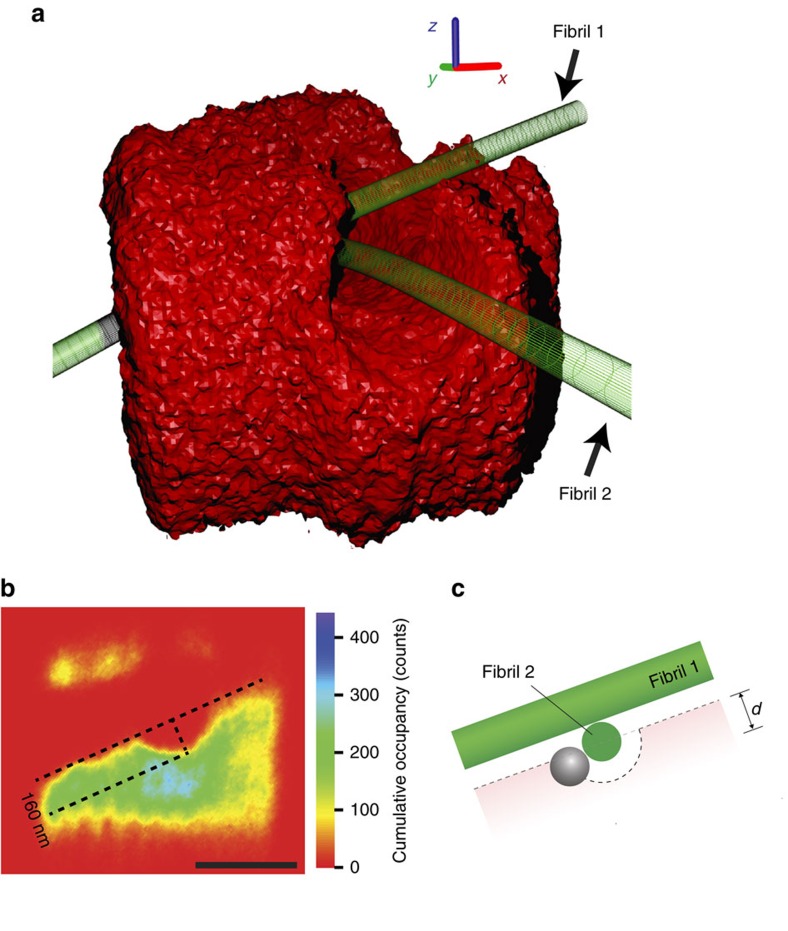
Submicroscopic structure of a junction of collagen fibrils. (**a**) Thermal Noise image of a junction of two collagen fibrils, which are sketched in green to guide the eye. Axis cues are 200 nm in length. For a view from multiple angles; see Supplementary Movie 1. (**b**) Slice through the cumulative occupancy perpendicular to fibril 2 at the position of fibril 1. The channel formed by fibril 1 is ∼160 nm above the channel formed by fibril 2 (Scale bar, 500 nm). This indicates that fibril 2 had a diameter of about 160 nm if the two fibrils lie on top of each other (**c**).

The most challenging situation for the correction algorithm is the crossing of two or more filaments. Figure 3 shows that such a crossing can be imaged with negligible distortion (for a view from multiple angles see Supplementary Movie 1). The filaments are locally straight and round, indicating that the offset correction works even under these extreme conditions. The filament crossing also allows for a probe particle independent consistency check of the filament diameters. Figure 3b shows a slice through the thermal noise image in Fig. 3a along fibril 1 and perpendicular to fibril 2. The histogram has a small indentation where the probe has to roll around fibril 2. The depth of this indentation is independent of the probe diameter (Fig. 3c), and we estimate that fibril 2 has a diameter of 160 nm.

**Figure 4:**
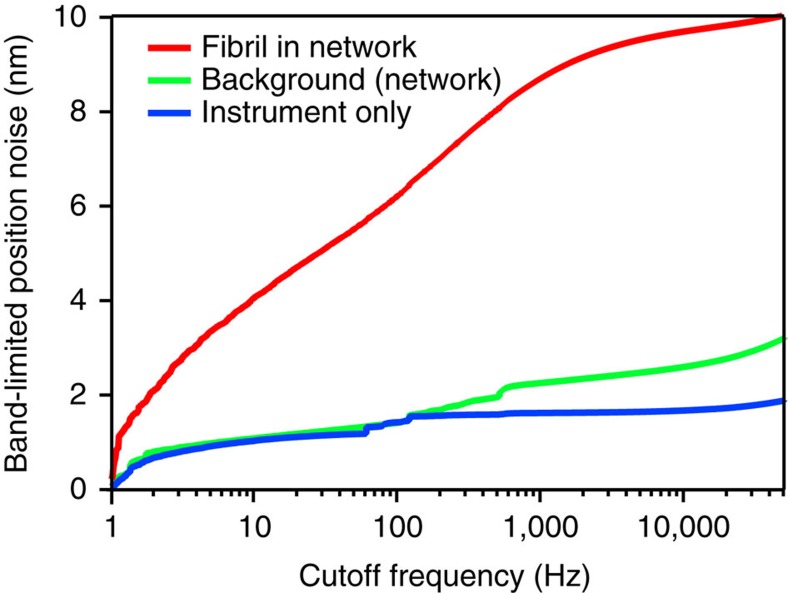
Magnitude of lateral fluctuations of a collagen fibril in a network. Most of the power of the fibril's lateral fluctuations is distributed over the acoustic frequency regime; the curve plateaus at high frequencies, as expected (red curve). The motion of the fibril can be easily separated from the background noise caused by the out-of-focus rest of the network (green curve) and from position noise of the instrument (blue curve). The position fluctuations were recorded with a sampling rate of 100 kHz at 1 MHz electronic bandwidth.

We demonstrate in the previous paragraph that the offset correction results in local geometries with negligible distortion even under difficult conditions. This is only possible if the material above and below the focal plane has a negligible influence on the detector signal. In addition, if any other filaments were located nearby, they would result in additional excluded volumes in the thermal noise image. Thus, we can assume that the detector offset signal originates solely from the imaged fibril. Hence our method opens a new opportunity to study the dynamics of individual collagen fibrils in a network. When the laser is focused on a fibril, any fluctuation measured corresponds to the transverse motion of the filament. The signal can be calibrated using the detector response measured in the offset scan (Supplementary Fig. 4). Figure 4 shows the s.d. of the transverse fluctuations for the fibril shown in Supplementary Fig. 4. The main contribution to the signal stems from the frequency interval from 1 Hz to 1 kHz. The slight increase at higher frequencies is caused by a combination of background (green line) and instrument noise (blue line).

The s.d. of the fluctuations in the relevant frequency range is clearly above the instrument noise but below 10 nm. The average value of the transverse fibril fluctuations was 7±2 nm for *N* random positions along fibrils in the network (*N*=11). This value is interesting for several reasons: it justifies the assumption we made for our offset correction, namely that the fibrils are rigid and do not significantly contribute to the fluctuations of the signal (see Methods). Further, it is the first quantitative measurement of transverse filament fluctuations for fibrils in a collagen network *in vitro*. This result supports the long-standing assumption that collagen networks are athermal, that is their transverse fluctuations are negligible for the elastic response of the network. In contrast, the mechanical response of actin filament networks is dominated by transverse thermal fluctuations^18^. Finally, this result underlines that even very rigid filaments confined in a network show transverse fluctuations on the order of 10 nm, which can be detected only with high precision and high-bandwidth methods.

## Discussion

We have demonstrated that thermal noise imaging can yield quantitative nanoscale images of soft-matter structures, and we have used the technique to quantify the dynamics of single fibrils in a network. Our method achieves a resolution better than 10 nm in localizing the axis of filaments, and an offset correction makes possible the determination of local geometry even in heterogeneous media. Our probe particles are larger than fluorescent probes and are not specifically attached to a target, but we gain bandwidth and precision, which are essential when investigating soft fluctuating structures. Because thermal noise imaging is not limited by labelling density or photobleaching, higher resolution can be achieved simply by recording more probe positions while confining the probe's diffusion to the same trapping volume.

Recently, it has been shown that integrating fluorescent probes into intracellular biopolymer networks can change the adhesion dynamics of cells^19^, and may alter the force-induced rearrangement dynamics of their cytoskeletons^20^. In contrast to fluorescence-based super-resolution techniques, thermal noise imaging avoids such labels, reducing the risk that the structure, mechanics and dynamics of the imaged soft matter are altered.

Probe particles for thermal noise imaging have to be added to the sample before imaging. This is also true for fluorescence microscopy, unless genetically expressed fluorescence labels are used. As with fluorescent probes, our probe particles can only reach volumes accessible by diffusion. Improvements in thermal noise imaging may narrow the gap in size between fluorescent probes and probe particles.

The resolution in thermal noise imaging depends on the bin width (typically 10 nm), the number of points in the position histogram (here 400,000), and the softness of the interaction potential between probe and structure. The probe's radius sets the smallest feature size that can be imaged (Supplementary Fig. 5). Reducing the diameter of the probe particle (here 190 nm) would increase both the spatial resolution and the imaging speed; however, smaller probe particles generate a smaller position signal, increase the relative background signal from other structures, and have a weaker trapping potential. The latter is required to force the probe particle to revisit the same volume repeatedly until it is sufficiently sampled. Small metallic nanoparticles have approximately sevenfold the trapping efficiency of equally sized polystyrene beads^21^ and feature an order of magnitude higher scattering cross-section^22^. Metallic particles as small as 10 nm have been stably trapped and tracked in a near-aberration free beam^21^. Small silicon particles feature trap stiffnesses comparable to metallic particles without the associated heating of the surrounding fluid and could also be a viable candidate for thermal noise imaging^23^. We have begun to evaluate the use of metallic particles as probes in thermal noise imaging and have stably trapped and tracked 100 nm diameter beads in biopolymer networks (data not shown). Despite the optical aberrations, that is, deformations of the wave-fronts of the trapping beam introduced by the network, we believe that the probe diameter can be further reduced to about 50 nm.

Collagen, in addition to being the most prominent protein in humans, constitutes an extreme example for a heterogeneous network of thick refractive filaments, which are most difficult to image without distortions. Here we recorded their three-dimensional images with a negligible amount of distortions by recording the offset signal that they cause and correcting the probe's position signal for this offset. In contrast, no such corrections were necessary for thermal noise images of microtubules, because we found the offset signal from the microtubule filament to be negligible compared with the signal caused by the probe. We also expect this to be true for most filament networks with filament thickness in between that of collagen fibrils and microtubules. As filaments get thinner, their effect on the position detection of the probe particle decreases rapidly despite an expected increase in transverse fluctuations. Thus, the results described here should be seen as evidence that thermal noise imaging is able to provide correct data even under extreme conditions (strong optical heterogeneity and thick sample) with most future applications being less demanding.

At first glance, the ability of thermal noise imaging to follow dynamic changes in a sample seems to be limited by the time required to record a complete image of at least one trapping volume. This, however, is not the case because a thermal noise image contains quantitative information about structural dynamics faster than the probe position distribution's integration time. LRO profiles change with the fluctuation of the imaged structure (Fig. 1f–h) and contain information on its thermal motion with a negligible amount of motion blur. Such LRO profiles can provide detailed information about the mechanics of the imaged structure when an adequate theoretical analysis is applied. In contrast, low-pass filtering distorts the structure's spatial probability distribution as measured by other super-resolution techniques, which results in a flawed extraction of the structure's mechanical properties (Supplementary Fig. 6). LRO profiles also reflect the potential energy between filament and probe in units of the thermal energy *k*_B_*T* if the filament can be considered rigid and the volume is visited sufficiently often. For a fluctuating filament the LRO is the potential of mean force felt by the probe.

Supplementary Figure 7 shows low-pass filtered thermal noise images of a microtubule. It illustrates how long integration times lead to wrong interpretation of the imaged structure. The original image was recorded with an integration time of 1 μs. When it is increased to 100 μs, an increase in the radius of the excluded volume is observed (Supplementary Fig. 8) and the number of position measurements decreases, accordingly making the isosurfaces noisier. This increase in radius is a result of low-pass filtering of the asymmetric radial position distribution of the probe: the particle cannot diffuse into the filament, so every collision with the filament leads to a displacement of the probe away from the microtubule. The motion-blurred position of the particle during the collisions is hence biased towards positions away from the filament, which in turn leads to an increased excluded volume. When the integration time *τ*_int_ is so long that the particle has sufficient time to diffuse around the filament, the radius starts to shrink (*τ*_int_=10 ms) and eventually the excluded volume completely disappears (*τ*_int_=40 ms). If our bandwidth were only on the order of tens of hertz, we would conclude that there is no filament and that the particle was located at the center of the optical trap, while it was actually excluded from this position. This effect gets stronger with decreasing particle radius underlining that high detector bandwidth is essential for high precision in localization of diffusing probes. But, even when the probe particle is not freely diffusing, our data show that structures that are considered very rigid fluctuate on a 10 nm and longer length scale unless they are immobilized on a very rigid substrate such as a microscope slide.

So far, thermal noise imaging uses one single probe particle for imaging. Multiple particles in parallel would significantly increase the imaging speed. In principle, this is possible if crosstalk in detecting different particles can be avoided or corrected for. Extension to two probe particles can be achieved by splitting laser polarization and forming two orthogonal optical traps^24^. Simultaneous imaging of more than two particles can be achieved by timeshared optical traps; however, true simultaneous multi-particle imaging requires not only the formation of multiple optical traps but also independent three-dimensional high-resolution and high-bandwidth detection that is currently achieved only for up to two probe particles.

Both super-resolution microscopy techniques and thermal noise imaging aim at determining the position of an object relative to other structures with nanometre precision, whereas thermal noise imaging provides a wealth of additional information. The measured probability distribution reports on the size and flexibility of structures and on the interaction potential between particle and scanned surface. Forces transmitted through biopolymer networks change the lateral fluctuations of individual filaments in the network: filaments under high tension fluctuate less than those under low tension. Hence, by measuring the lateral fluctuations of filaments, thermal noise imaging also reports on the force transmitted through a structure. This information is essential for describing the mechanics of force transmission through networks which can no longer be considered homogenous on the length scale of interest, for example, a cell migrating through a collagen network. So far, forces are calculated from displacements of markers in the network assuming a linear force–displacement relationship, which does not account for the known highly non-linear force response of connective tissue. The non-linear response seems to arise from local non-affine deformations such as fibre buckling, straightening or stretching, which only recently have been taken into account in modelling^25^,^26^. The novel type of microscopy described here will enable us to observe such local non-affine deformations and it will allow us to monitor their mechanical consequences with sub-diffraction limited resolution. This is a result of not only our ability to measure the mean position of fluctuating objects but also of being able to measure their fluctuations around the average correctly. In general, thermal noise imaging opens up new possibilities to study the relationship between structure and dynamics of soft matter, including cells, on a length and time scale that so far has not been accessible. In this regard, thermal noise imaging is complementary to current super-resolution microscopies and future instruments might use both imaging modes.

## Methods

### Passivation of probe particles

Fluorescent polystyrene microspheres (sun coast yellow, diameter 190±30 nm, Bangs Laboratories, IN, USA) were passivated against adhesion to collagen fibrils by coating with poloxamer 407 (16758, Sigma Aldrich, MO, USA), a block co-polymer consisting of a central hydrophobic block of polypropylene glycol (PPG, 67 repeat units), flanked on each side by a hydrophilic polyethylene glycol block (PEG, 98 repeat units)^27^. Tracer particles were incubated at room temperature at least overnight in a solution of 2 mg ml^−1^ poloxamer 407 in PBS. From this stock solution the particles were further diluted into PBS for each experiment. Poloxamer 407 self assembles as a brush on the surface of the polystyrene beads^27^, with the hydrophobic PPG block adhering to the particle, while the hydrophilic PEG blocks form coils pointing radially outwards.

### High bandwidth and high precision trapping and tracking

All data were acquired using a custom-built photonic force microscope (PFM) featuring an ultra-stable optical trap coupled to a three-dimensional, high-precision and high-bandwidth position detector. Our PFM is capable of tracking the position of a 190 nm diameter polystyrene probe particle with a precision of 1.5 nm laterally and 7 nm along the optical axis at a bandwidth of 1 MHz. The beam of a 1064, nm laser (Mephisto 500 mW, Coherent, CA, USA) was expanded and focused through a water immersion objective lens (UPlanSApo 60xW, Olympus, Tokyo, Japan), forming an optical trap at its focus. The sample was mounted on a three-dimensional nano-positioning stage (Nano-View/M375HS, Mad City Labs, WI, USA), which allowed its motion relative to the stationary optical trap. Forward scattered light from a trapped probe particle together with unscattered light of the trapping beam was collected by a condenser lens and projected onto a quadrant photodiode (G6849, Hamamatsu Corporation, NJ, USA), where the two waves interfered. The voltage outputs of the diode's quadrants were amplified by custom-built low noise differential amplifiers (SA500, Oeffner MSR, Plankstadt, Germany) and can be related to the *x*, *y* and *z* position of the particle relative to the center of the optical trap^28^,^29^. Typical spring constants of the potential confining the tracer particle were *k*_*x*_=1.5 pN μm^−1^, *k*_*y*_=1.0 pN μm^−1^ and *k*_*z*_=0.15 pN μm^−1^. The corresponding autocorrelation times of the tracer particle's diffusion were *τ*_*x*_=1.1 ms, *τ*_*y*_=1.7 ms and *τ*_*z*_=11 ms.

### Background correction for probe position signal

In absence of optically heterogeneous material, the position of the probe is determined by reading out the interference of light forward scattered by the probe with light of the trapping beam on the PFM's quadrant photodiode. If the probe as well as the collagen fibril are close to the focus, both light scattered by the probe and by the fibril will strike the detector. The output signal therefore no longer correctly reflects the probe's position and must be corrected for the fibril contributions. It can be shown^30^,^31^ that to first order approximation the total detector signals along the *x*, *y* and *z* directions *S*_α_ (**b**_p_, **b**_f_), are the sum of the signals on the detector caused by the fibril in absence of the probe *S*_*α,* fibril_ (**b**_f_), and the signal caused by the particle in absence of the fibril *S*_*α,*probe_ (**b**_p_) (*α*=*x, y, z* and **b**_p_ and **b**_f_ are the position vectors of the particle and fibril with respect to the focus, Supplementary Fig. 3)


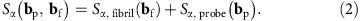


As shown in this work, collagen networks are athermal to good approximation, and we may assume that the positions of the fibrils **b**_f_ and their signals *S*_*α,* fibril_ (**b**_f_) are constant. The signal *S*_*α*_, excluding the constant offset, measured at a certain grid position (*x*_grid_*, y*_grid_*, z*_grid_) is hence only a function of the probe's position **b**_p_ and equal to the probe's position signal *S*_*α,* probe_ (**b**_p_), plus a constant offset determined by the network's structure around the specific grid position at which the data are acquired. If the offsets are known for each grid position, the particle's position can be calculated from the measured signals as


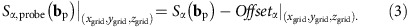


For all the data shown in this work, we first acquired the total signals *S*_*α*_(**b**_p_) at each grid position of interest. Subsequently, we released the probe from the optical trap and revisited all scanned grid positions to acquire 200 ms long time traces of the offset signals *S*_*α,* fibril_ (**b**_f_), the mean of which give the offsets *Offset*_*α*_ at each grid position. We then computed the particle position signals *S*_*α,* probe_ (**b**_p_) as explained above.

Collagen networks are only approximately athermal and the offset signals *S*_*α,* fibril_ (**b**_f_) are not truly constant but contain information on the fibril's transversal motion (see main text). The s.d. of *S*_*α,* fibril_ (**b**_f_) calibrated with the probe position detector's sensitivity yields additional probe position uncertainty of 3, 4 and 9 nm along *x*, *y* and *z*, respectively, caused by the fibril's fluctuation.

### Calibration of the position detector

The three-dimensional detector's non-linear response *S*_*α,* probe_ (**b**_p_) was linearized and calibrated *in situ* as described by Tischer *et al*.^29^ The linear order coefficient of the resulting polynomial function, relating signal to displacement, is commonly referred to as the detector's sensitivity.

### Polymerization of microtubules

Microtubules were grown by suspending 4 μg of unlabelled tubulin and 0.8 μg of rhodamine labelled tubulin (T240 and T590M, respectively, Cytoskeleton, CO, USA) in 25 μl BRB80 (80 mM PIPES, 1 mM EGTA, 2 mM MgCl_2_, pH 6.8) supplemented with 1 mM guanosine triphosphate (GTP) and incubating at 37 °C for 10–30 min, depending on the desired microtubule length. After polymerization, microtubules were stabilized by resuspension in BRB80 supplemented with 20 μM taxol.

### Single microtubule assay

Microtubules were spanned in random directions over a holey carbon film (hole width: 7 μm, hole periodicity: 9 μm) on a copper electron microscopy grid (spacing of the copper grid: 127 μm; S 7/2, Quantifoil, Electron Microscopy Sciences, PA, USA). The 20 μm thick copper grid was glued to a glass coverslip using a biochemically inert, solvent-free silicone glue (Elastosil N10, Wacker, Germany). The copper's thickness provides sufficient lift to avoid any hydrodynamic coupling between the glass coverslip and the microtubules or tracer particles.

Microtubules must be grafted to the holey carbon film strongly so that both end points (static microtubule) or one end point (fluctuating microtubule) and the tangent of those points are fixed. This was achieved by exposing the grid for 2 s to an oxygen plasma and then coating the holey carbon film by 50% poly-L-lysine (PLL) in deionized water. PLL has a strong non-specific interaction with microtubules and is commonly used to immobilize them on surfaces^32^. The coverslip with the glued, plasma cleaned grid was then quickly assembled to a sample chamber by adding a metal spacer, and after the next steps, a top coverslip.

The carbon film was then immediately coated with 50 μl of PLL solution and incubated for 20 min at room temperature. In typical assays, the PLL solution is allowed to dry on the surface onto which the microtubules are to be adhered. This was not possible here, since the air–liquid interface of the drying PLL solution destroyed the carbon film on the grid by surface tension. Instead, the PLL solution was flushed off the grid with deionized water.

At this point the top coverslip was added to the sample chamber. Fluid inside the chamber could be exchanged through small channels in the metal spacer: 200 μl of BRB80 buffer to displace the deionized water, and subsequently 200 μl of microtubules in BRB80 were flushed into the chamber. The microtubules were allowed to adhere to the PLL coated carbon film for several minutes. Unattached microtubules were then removed from the sample chamber by a flush with 200 μl of a solution containing probe particles and an oxygen scavenging system consisting of 50 U ml^−1^ glucose oxidase, 500 U ml^−1^ catalase and 12.5 mM glucose in BRB80 supplemented with 20 μM taxol.

### Computing radial LRO of a thermal noise imaged microtubule

To test the reproducibility of thermal noise imaging we imaged a double-confined microtubule 33 times in a row ∼2 μm away from the support. For the fluctuating microtubule, we acquired the data 2.8 μm from the solid support. A probe particle was held in a weak optical trap and was allowed to diffuse around the microtubule, which intersected the trapping volume and was supported on both of its ends. For each measurement, the particle's motion was recorded for 4 s with an electronic bandwidth of 1 MHz and a sampling rate of 100 kHz. Position histograms were calculated from the 400,000 measured positions. We then projected the three-dimensional position histograms along the microtubule's axis into two-dimensional histograms, and computed the corresponding two-dimensional LROs (see main text and Fig. 1e,f,g). Each two-dimensional LRO was fitted with a two-dimensional Gaussian function to find its center. We then computed the radial LRO from each two-dimensional LRO by averaging the data on concentric circles as described by Zhang *et al*.^33^ The LRO has values of positive infinity in the excluded volume. To account for rare events in which the particle visited voxels close to the excluded volume only once (or very few times) values of infinity must be appropriately dealt with during the radial averaging, we empirically chose to set the value of the radial LRO for a certain radius to infinity if at least 30% of the two-dimensional LRO's values at this radius were equal to infinity. Conversely, our threshold ensures that we only set the radial LRO to a numerical value if the particle could visit the corresponding radial extension at least 70% of the time. The resulting radial LROs were then averaged and the s.d. of this average was computed for each radial position (Fig. 1h).

### Preparation of collagen networks

Collagen networks were polymerized without fluorescence labels following a procedure established by Mickel *et al*.^16^ In brief, acid-soluble rat-tail tendon collagen (type I, Collagen R, 354236, BD Biosciences, NJ, USA) and bovine-dermis collagen (type I, Collagen G, 354231, BD Biosciences, NJ, USA) were mixed at relative concentrations of 1:2. The mixture was then diluted to a total collagen concentration of 2.4 mg ml^−1^ by adding equal parts of 10 × DMEM (D2429, Sigma Aldrich, MO, USA) and 0.27 M NaHCO_3_. To induce gel polymerization, the pH of the solution was raised to pH 10 using 1 M NaOH. All components were kept on ice during mixing. The mixture was then quickly pipetted into a preassembled sample chamber consisting of a glass coverslip attached by vacuum grease to a metal washer (Supplementary Fig. 9), and left to polymerize for >45 min at 37 °C in a 5% CO_2_ atmosphere. Polymerizing the network inside the sample chamber ensures its attachment to the coverslip, which is a prerequisite for a mechanically stable assay. Networks were between 500 μm and 1 mm thick. After polymerization, the gel was gently rinsed with 1 ml of 1 × PBS. Care was taken to never let the network dry out.

### Collagen sample assembly

After polymerization of the collagen network, the sample chamber was closed by attachment of a top coverslip (Supplementary Fig. 9). Passivated tracer particles in PBS were flushed into the chamber through channels in the metal spacer. The laminar flow of this flush deposits particles predominantly above, but not inside, the collagen network. The sample was then incubated for at least 2 h to allow the tracer particles to diffuse deep into the gel. At this point, the sample chamber was mounted onto the PFM.

### Scanning strategy for long-range thermal noise images

The exploration of an individual trapping volume by the probe particle is driven by thermal forces and is thus limited by diffusion. To ensure that voxels displaced from the center of the trap as far as 150 nm laterally and 450 nm axially were reliably visited by the probe particle the probe's position was tracked for 4 s for each individual trapping volume, which is the integration time of each individual thermal noise image. A volume of approximately 1 μm × 1 μm × 1 μm can be imaged by acquiring an individual position histogram measurement at each grid point on a 10 × 10 × 2 grid (compare red dots in Supplementary Fig. 2a spaced 100 nm laterally and 300 nm axially). Much of the imaged space is empty and its exploration by the particle does not yield information on the network's filaments. As long as no filaments are present at a certain grid point, it is not necessary to acquire a position histogram at its position. Acquiring images only at grid positions close to filaments reduces the total imaging time significantly. Thus, a more sophisticated scanning strategy was implemented to reduce the total acquisition time.

To find the positions close to filaments a predefined grid size was chosen and the probe particle was rapidly raster scanned through the sample volume along the grid. At each position, 100 ms long time traces of raw, uncalibrated position signals were acquired. Using the detector's sensitivity, this signal was related to the probe's position (albeit not yet corrected for nonlinearities in the detector response), plus an unknown position offset since the detector has not yet been corrected for the signal offsets introduced by the light scattered by the network. From these time series the s.d., *σ*_*α*_, were estimated and compared to the reference s.d. of diffusion in an empty trapping volume, given by *σ*_*α,*ref_=(*k*_B_ *T/k*_*α*_)^1/2^, where *α*=*x*, *y*, *z*, *k*_*α*_ are the spring constants of the optical potential, *k*_B_ is the Boltzmann constant and *T* is the temperature. If a filament is present in the trapping volume, the particle can no longer explore the entire volume, and the s.d. of the particle's motion along at least one axis is expected to decrease (Supplementary Fig. 10). A decrease of at least one of the s.d. to *σ*_*α*_*<*0.4 *σ*_*α,*ref_ was empirically found to be a reliable measure to determine whether fibrils were close to a given grid position. Position histograms were acquired at all grid positions that fulfilled this condition, and at their nearest neighbours.

The described strategy is a feedback mechanism on the dwell time at a certain grid position: if no filament is present, the grid position is not revisited for the acquisition of a position histogram, and the full dwell time spent at it is 100 ms. If the probe interacts with a filament, the position histogram is measured by acquiring a 4 s long time trace of the particle's diffusion, and the total dwell time at the grid position is 4.1 s. This approach can significantly decrease total acquisition times depending on the amount of empty space in the imaging volume. For example, for the image shown in Supplementary Fig. 4 the total data acquisition time was reduced from 7.3 min to 4.4 min.

### Calculation of the band-limited position noise

The band-limited position noise of the collagen fibril fluctuations (Fig. 4) was calculated by computing the power spectral density (PSD) of a 1 min long time trace of fibril positions sampled at 100 kHz at an electronic bandwidth of 1 MHz. The PSD was then integrated from 1 Hz to the cutoff frequency as described by Kochanczyk *et al*.^34^, yielding the variance of the position noise in the corresponding frequency band. We then computed the band-limited s.d. from this variance.

To determine the background noise contributed by out of focus filaments, we positioned the focus of the trapping beam in a pore of the network, ∼1.3 μm from the fibril discussed above, and recorded the beam's signal for 1 min at 100 kHz at an electronic bandwidth of 1 MHz. We calibrated this signal with the fibril's sensitivity and computed its band-limited s.d.

The noise floor of the instrument was determined by immobilizing a probe particle onto a glass slide and sampling its position for 1 min at 100 kHz with 1 μs integration time for each data point. The band-limited s.d. of the immobilized bead was computed as explained above, and gives the limit of our instrument's precision.

### Data analysis and visualization of thermal noise images

The acquired data were analysed using custom software written in Labview (National Instruments, TX, USA) and Igor Pro (Wavemetrics, OR, USA). Thermal noise images were visualized using VisIt^35^ and Blender (http://www.blender.org).

### Data availability

Data supporting Figs 1–4, S4 and S7 have been deposited in Dryad (doi:10.5061/dryad.c2j90). Other data are available from the corresponding author upon request.

## Additional information

**How to cite this article:** Bartsch, T. F. *et al*. Nanoscopic imaging of thick heterogeneous soft-matter structures in aqueous solution. *Nat. Commun.* 7:12729 doi: 10.1038/ncomms12729 (2016).

## Supplementary Material

Supplementary Information Supplementary Figure 1-10, Supplementary References

Supplementary Video The video shows the thermal noise image of a junction of two collagen fibrils as displayed in Figure 3, from multiple angles. The positions and orientations of the filaments are sketched as wireframes to guide the eye. Axis cues are 200 nm in length and the isosurface is shown for an occupancy of 10 counts. In the far right corner, separated from the rest of the image, one can see the image of an empty trapping volume. This is the position where the raster scan was started and which was used to calibrate the position detector.
