# Supplementary material for: Nanoscopic imaging of thick heterogeneous soft-matter structures in aqueous solution
Source: Nat Commun. 2016 Sep 6;7:12729. doi: 10.1038/ncomms12729 (PMC5025867; doi:10.1038/ncomms12729)
Supplement: Supplementary Information — Supplementary Figure 1-10, Supplementary References [file ncomms12729-s1.pdf]

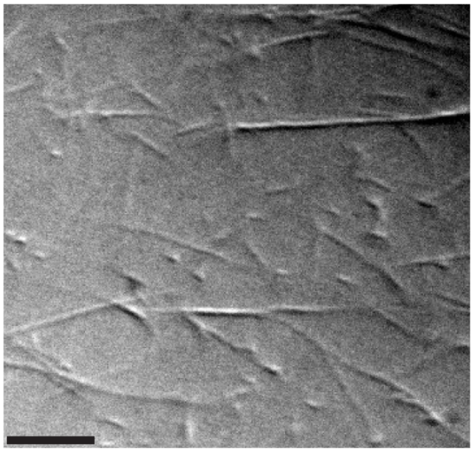

Supplementary Figure 1. Differential-interference contrast image of a collagen network. The large contrast of the fibrils indicates their high optical density compared to the surrounding medium. Fibrils with varying diameters can be seen forming a network with a mesh size of a few micrometers (see main text and Methods). Experimental conditions were as described in the Methods section. Scale bar: 5 μm.

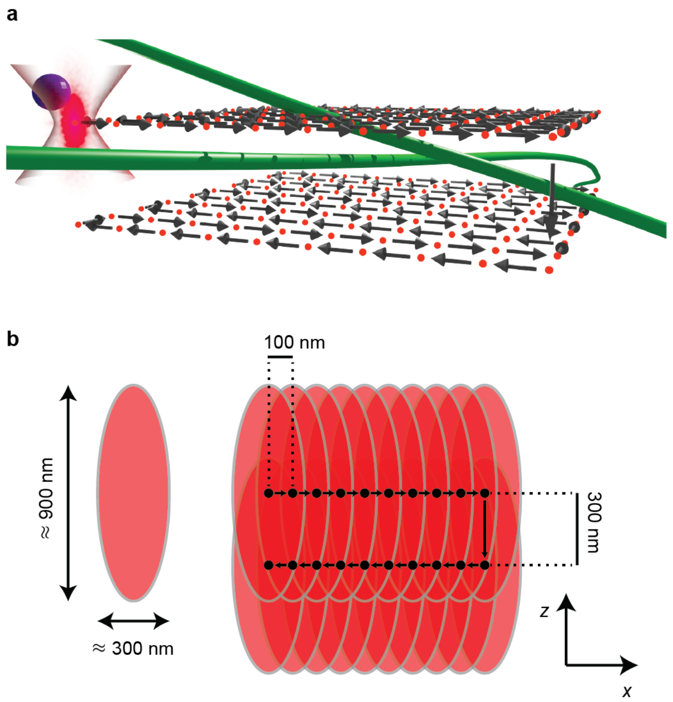

Supplementary Figure 2. Long-range thermal noise imaging in a polymer network. a – Position histograms of the confined particle's motion are acquired centered around positions spaced by 100 nm laterally and 300 nm axially. Two collagen fibrils (green) are drawn to indicate the network. **b** – Illustration of the overlap of individual occupancy measurements. A position histogram for the entire grid is populated from the individual occupancies by summing up counts of overlapping voxels. This cumulative occupancy, or global thermal noise image, that spans the entire grid is typically displayed by occupancy isosurfaces.

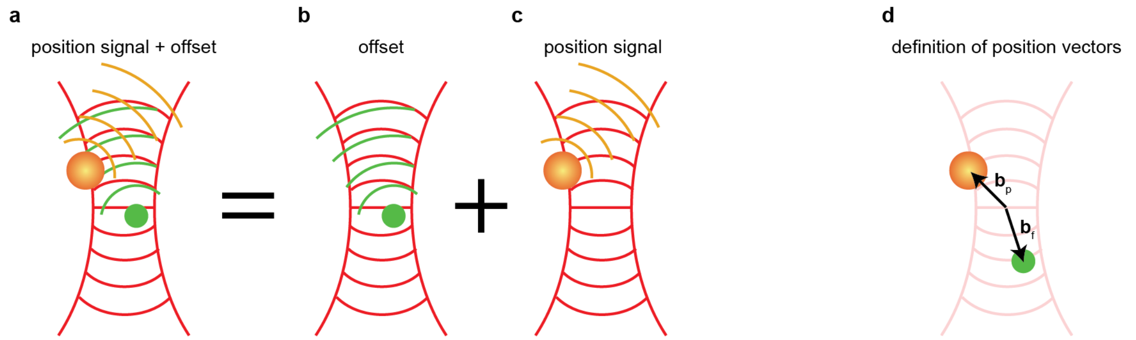

Supplementary Figure 3. Correcting the position signal for network scattered contributions. To first order approximation, the intensity striking the detector (**a**) may be approximated as the sum of the signal caused by the network alone (**b**), plus the position signal of the particle in absence of the network (**c**). **a** is acquired by reading out the detector while the particle is trapped. **b** can be independently measured after releasing the particle from the trap. The actual position signal (**c**) can then be computed[1] **d** – Definition of the particle and filament position vectors.

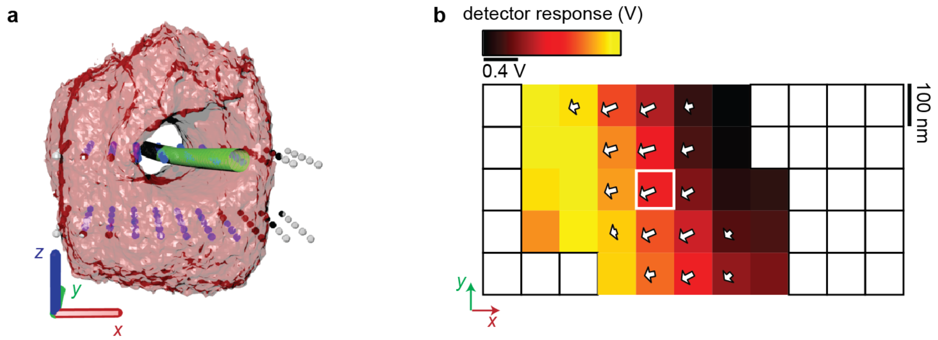

Supplementary Figure 4. Calibration of the position sensor for lateral fibril fluctuations. a – Thermal noise image of a collagen fibril in a network. Spheres mark the grid positions of the scan, spaced as described in Supplementary Figure 2. Data was only acquired at grid positions in close proximity to the collagen fibril (blue spheres). The collagen fibril wireframe is shown to guide the eye. Axis cues are 200 nm in length and the isosurface is plotted at 10 counts. **b** – Average detector response (squares) along the **x**-direction in the absence of the probe, for each grid position at which data was acquired on the upper *x*-*y* plane of the grid shown in **a**. Iso-response lines form an angle of approximately 105 degrees with the *x*-axis. The same angle was independently found for the orientation of the fibril from the thermal noise image in **a**, a strong indication that the observed response is due to light scattered by the fibril. The gradient of the detector response (arrows) is the detector's sensitivity for displacements of the fibril. The arrow in the white-outlined pixel corresponds to a sensitivity of 4.6 mV nm^{-1} for transversal motion.

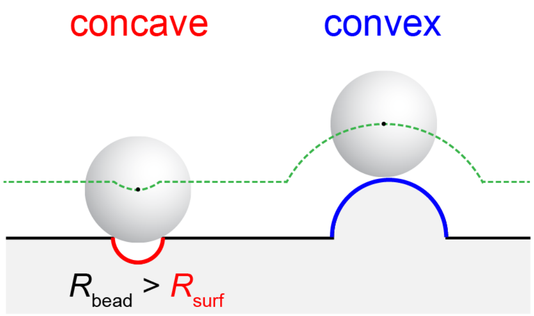

Supplementary Figure 5. Thermal noise imaging of concave and convex features. Concave features can only be accurately imaged if the probe particle can fully penetrate into the feature, that is concave structures with $R_{surf} > R_{bead}$ can be imaged without loss of information. No such restriction exists for convex features.

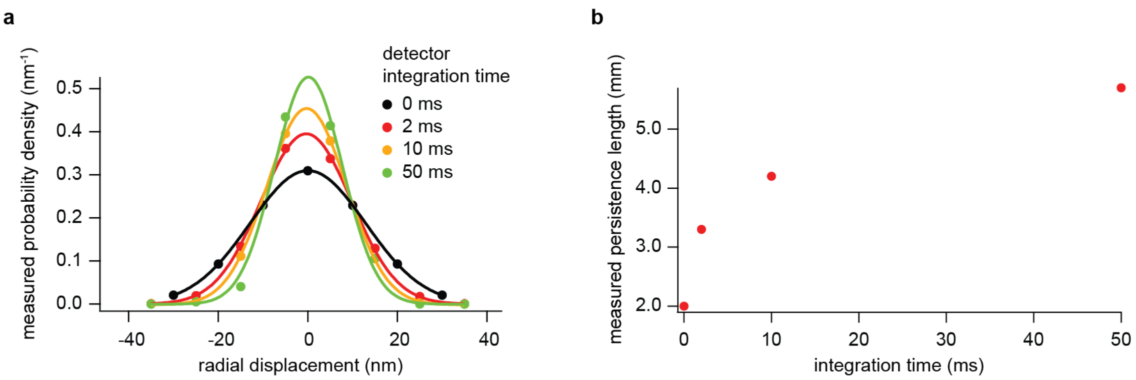

Supplementary Figure 6. Simulation of the lateral fluctuations of a microtubule with different detector integration times. The lateral fluctuations of a 7.5 µm long microtubule (persistence length l_p = 2 mm) supported on one end and free to fluctuate on the other end were simulated by Brownian Dynamics with 1 µs temporal resolution, taking the first four bending modes of the filament into account. Time traces of radial filament displacements were analyzed at a distance s = 1 µm from the rigid support and low-pass filtered with integration times of 2 ms, 10 ms and 50 ms. **a** – Spatial position histograms of radial displacements for the different extents of low-pass filtering. As the integration time increases the width of the measured distributions shrinks. The black curve shows the analytical Gaussian fluctuation distribution with a variance $\text{var}(r) = s^3/(3\ l_p)$ (see reference 2). **b** – Typical experiments measure the filament's lateral fluctuations and find its persistence length from the width of the position distribution. The larger the integration time, the smaller the measured width of the distribution and the longer the measured persistence length. In order to extract accurate mechanical properties from such measurements a detector with a short integration time must be used. Microtubules were treated as cantilevered beams whose shape fluctuations decompose into a set of bending modes.[3] We simulated each filament's shape fluctuations by considering its first four bending modes and letting the mode amplitudes undergo random walks in their confining harmonic potentials defined by their stiffness $k_i = \gamma/\tau_i$, where γ is the drag perpendicular to the microtubule and τ_i is the autocorrelation time of the i-th mode.[3]

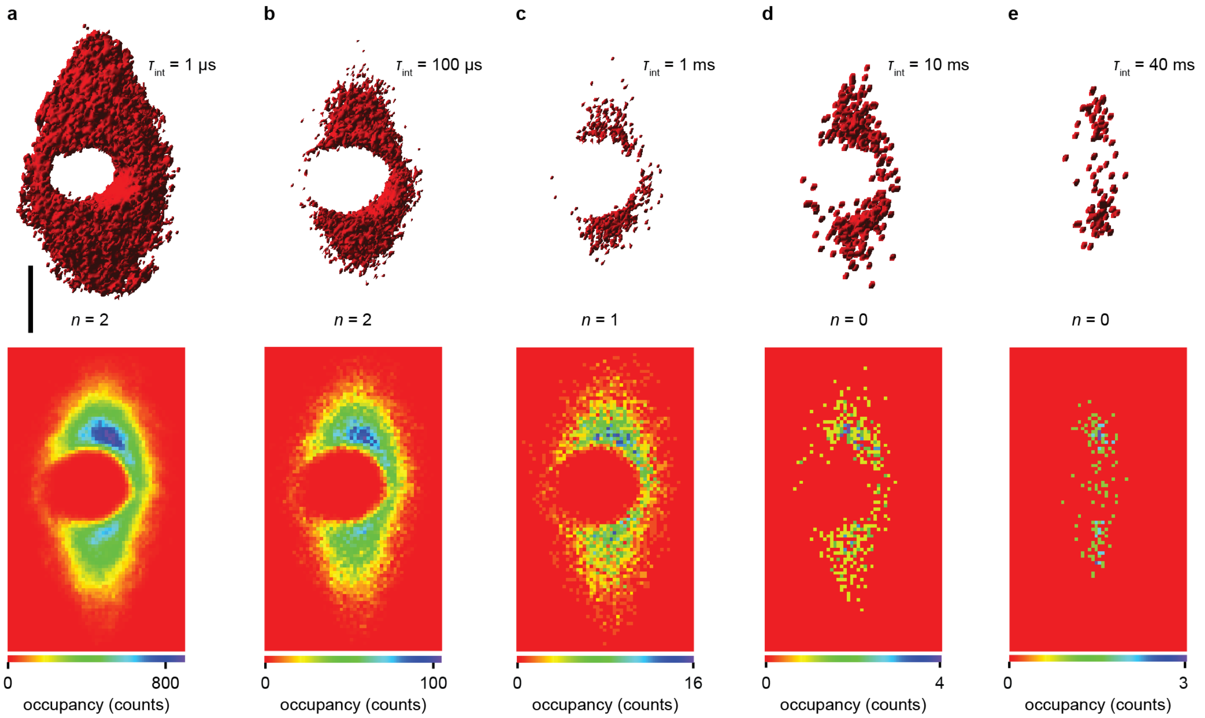

Supplementary Figure 7. **Distortion of the imaged local geometry as a result of low-pass filtered position data, here for the example of a thermal noise image of a microtubule.** The upper row shows isosurfaces (red) of probe-position histograms, drawn at *n* counts for different position detector integration times. The data were calculated from the original experimental position data recorded with 1 μs integration time (shown in panel **a**). The longest integration time corresponds to a typical integration time in STORM or PALM. Below are respective two-dimensional projections of the probe-position histogram along the filament axis, depicting the occupancy as a heat map. **a** – The original thermal noise image of a microtubule, as shown in Figure 1, recorded with 1 μs second integration time. The isosurface for occupancy count of 2 shows a cylindrical excluded volume. **b** – The integration time is increased to 100 μs, which leads to a decrease in the number of position measurements. **c** – An integration time of 1 ms leads to a further decrease in position measurements. The isosurface becomes noisier and the occupancy count had to be reduced to $n = 1$ to still result in a connected surface. **d** – For the integration time set to 10 ms, the isosurface count had to be lowered to $n = 0$, which now encloses only individual data points in three dimensional space. **e** – The excluded volume disappears completely at an integration time of 40 ms and one could conclude that the particle

visited the middle of the trapping volume, which is not possible due to steric hindrance of the microtubule. Scale bar: 200 nm.

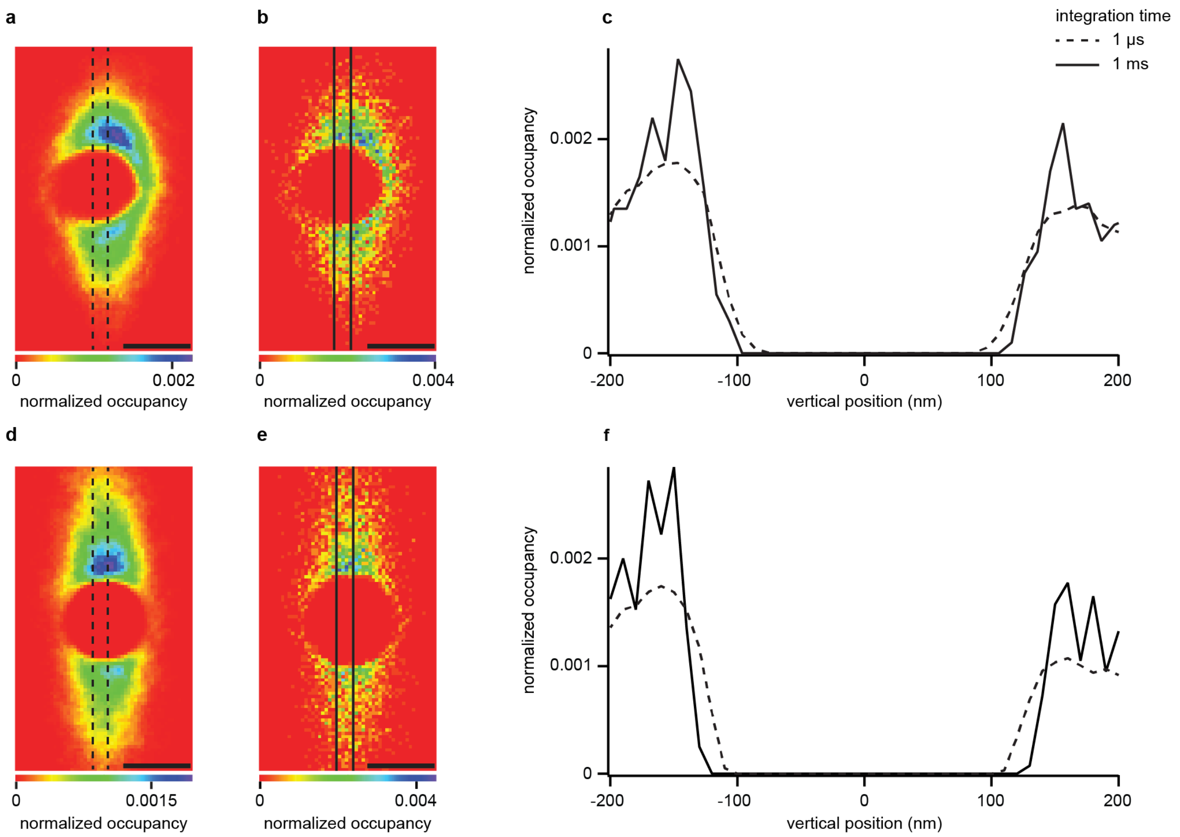

Supplementary Figure 8. Low-pass filtering increases the diameter of excluded volumes in thermal noise imaging. As shown in Supplementary Figure 7 the local geometry of thermal noise images becomes distorted with increasing integration time. Here we compare the two-dimensional projections of the probe-position histogram along the filament axis normalized with the total position count. **a** – Experimental realization at full bandwidth of 1 MHz (same data set as Supplementary Fig. 7, a). **b** – Same data as **a** but low-pass filtered to a bandwidth of 1 kHz (compare Supplementary Fig. 7, b). **c** – Vertical line-profiles in the full bandwidth case (averaged between dashed lines) and reduced bandwidth (averaged between solid lines) show a diameter increase of the excluded volume of approximately 71 nm. **d, e** – Brownian dynamics simulations of the same scenarios as **a** and **b**. **f** – The simulations show an increase of 60 nm in the diameter of the excluded volume, in good agreement with the experimental result. Scale bars: 200 nm.

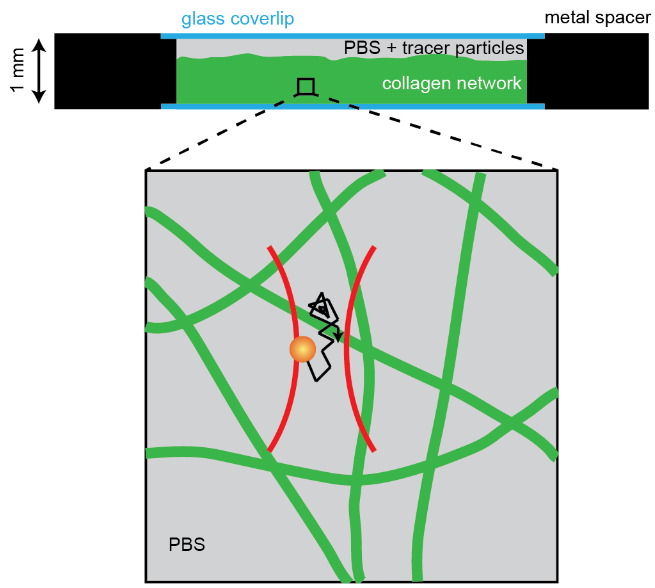

Supplementary Figure 9. Collagen network assay. A collagen network is polymerized inside a sample chamber, after which probe particles in PBS solution are added. Probe particles (orange) in the network (green) can be optically trapped (red beam) and thermal noise images can be acquired.

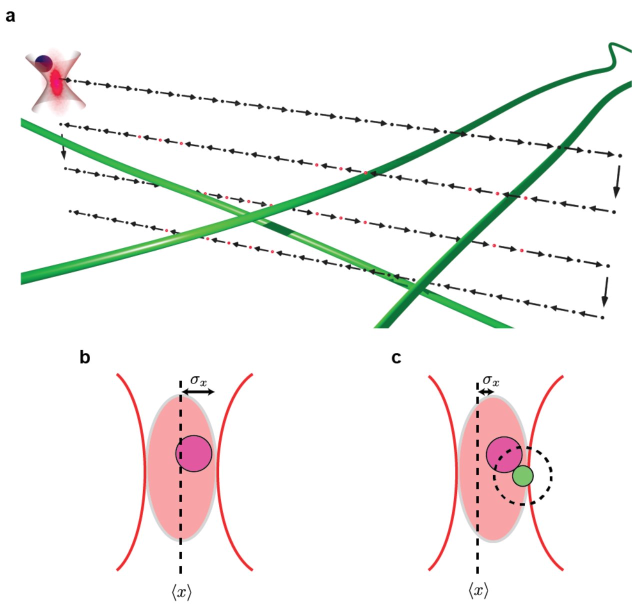

Supplementary Figure 10. Raster scanning strategy with feedback. a – Collagen fibrils are detected by a rapid scan of the trapped particle through the network. The presence of fibrils is detected by the change of the standard deviation of the tracer particle's thermal motion when a fibril is present (compare **b** and **c**). Probe-position histograms are only acquired at positions close to the filaments (red grid points in **a**), eliminating thermal noise imaging of empty space.

Supplementary References

1. Bartsch, T. F. Submicroscopic Characterization of Biopolymer Networks in Solution by Thermal Noise Imaging. PhD thesis, Univ. of Texas at Austin (2013).
2. Pampaloni, F. *et al.* Thermal Fluctuations of Grafted Microtubules Provide Evidence of a Length-Dependent Persistence Length. *PNAS* **103,** 10248 – 10253 (2006).
3. Janson, M. E. & Dogterom M. A Bending Analysis for Growing Microtubules: Evidence for a Velocity-Dependent Rigidity. *Biophys J* **87,** 2723 – 2736 (2004).
